# Analyzing magnetic bead QuantiGene® Plex 2.0 gene expression data in high throughput mode using QGprofiler

**DOI:** 10.1186/s12859-019-2975-2

**Published:** 2019-07-08

**Authors:** Bie Verbist, Eva Adriaensen, Vikki Keersmaekers, Dea Putri, Marjolein Crabbe, Maarten Derks, Rytis Bagdziunas, Griet Laenen, Hans De Wolf

**Affiliations:** 1 Janssen R&D, TMEDS, Turnhoutseweg 30, 2340 Beerse, BE Belgium; 2 Karel de Grote Hogeschool, Groenplaats, 2020 Antwerpen, BE Belgium; 3 Janssen R&D, Discovery Biology, Oncology Heme, Turnhoutseweg 30, 2340 Beerse, BE Belgium; 4 Dataroots, Tiensevest 132, 3000 Leuven, BE Belgium; 5 Open Analytics, Jupiterstraat 20, 2600 Antwerpen, BE Belgium; 6 Janssen R&D, DS, Turnhoutseweg 30, 2340 Beerse, BE Belgium

**Keywords:** Quantigene® Plex 2.0, High throughput transcriptomics, Drug discovery

## Abstract

**Background:**

The QuantiGene® Plex 2.0 platform (ThermoFisher Scientific) combines bDNA with the Luminex/xMAP magnetic bead capturing technology to assess differential gene expression in a compound exposure setting. This technology allows multiplexing in a single well of a 96 or 384 multi-well plate and can thus be used in high throughput drug discovery mode. Data interpretation follows a three-step normalization/transformation flow in which raw median fluorescent gene signals are transformed to fold change values with the use of proper housekeeping genes and negative controls. Clear instructions on how to assess the data quality and tools to perform this analysis in high throughput mode are, however, currently lacking.

**Results:**

In this paper we introduce QGprofiler, an open source R based shiny application. QGprofiler allows for proper QuantiGene® Plex 2.0 assay optimization, choice of housekeeping genes and data pre-processing up to fold change, including appropriate QC metrics. In addition, QGprofiler allows for an Akaike information criterion based dose response fold change model selection and has a built-in tool to detect the cytotoxic potential of compounds evaluated in a high throughput screening campaign.

**Conclusion:**

QGprofiler is a user friendly, open source available R based shiny application, which is developed to support drug discovery campaigns. In this context, entire compound libraries/series can be tested in dose response against a gene signature of choice in search for new disease relevant chemical entities. QGprofiler is available at: https://qgprofiler.openanalytics.eu/app/QGprofiler

**Electronic supplementary material:**

The online version of this article (10.1186/s12859-019-2975-2) contains supplementary material, which is available to authorized users.

## Background

Gene expression, through the quantification of mRNA is commonly used in biomedical research for patient diagnostics and/or therapeutics [1–3]. The quantification of mRNA is routinely performed using real-time quantitative PCR (RT-qPCR), measuring gene expression levels in a highly sensitive and specific manner [4]. However, there are limitations to this technique, which relate to the need for RNA extraction and the enzymatic based reverse transcription and target mRNA amplification steps which are prone to errors [5, 6].

Branched chain DNA (bDNA) technology, in which the signal and not the mRNA target sequence is amplified, provides a non-enzymatic alternative to qPCR [7–9]. The QuantiGene® Plex 2.0 platform (ThermoFisher Scientific) combines bDNA with the Luminex/xMAP magnetic bead capturing technology. This platform does not require an RNA extraction step, as it measures mRNA levels directly from cultured cells [10], cell lysates [11], tissue homogenates [12], formalin-fixed tissues [13], to name only a few starting points. The amplification of the signal depends on the cooperative hybridization between the target mRNA and three oligonucleotide probes. These probes are capture extenders, label extenders and blocking probes, whose sequences depend on the mRNA target sequence [11]. The hybridized mRNA target sequence is immobilized on the bead via a capture probe that links the bead with parts of the capture extender sequence, which provides the specificity of the signal [9]. The signal is subsequently amplified by adding a pre-amplifier sequence, which partly overlaps with the label extender sequence, and by several amplifiers, which generate the branched DNA structure and harbor hybridization sites for biotinylated label probes [11]. The biotinylated bDNA binds streptavidin conjugated R-phycoerythrin (SAPE) resulting in a raw fluorescent signal that is proportional to the hybridized mRNA quantity [7, 9, 14]. Besides the SAPE fluorescent signal, the Luminex reader also detects the internal dye of the individual beads allowing for differentiation between target specific beads. The Luminex/xMAP magnetic bead capturing technology allows for multiplexing in a single well of a 94 or 384 multi-well plate and is thus able to quantify the expression of a series of genes in a high throughput mode [11]. As such, the QuantiGene® Plex 2.0 platform (ThermoFisher Scientific) does not only offer the possibility to quantify mRNA levels in the context of patient diagnostics and/or therapeutics but can also be used in a high throughput drug discovery setting. Indeed, entire compound libraries could be tested, in single dose or dose response, against disease specific gene signatures in search for new disease relevant chemical starting points [10]. However, a proper data analysis framework should be put in place to analyze mRNA levels in high throughput mode.

The data analysis flow proposed by ThermoFisher Scientific is relatively straightforward and aims to translate and normalize gene expression, in the linear range of the assay, to fold change (FC) values, reducing signal variability due to sample preparation, sample input variability and/or overall well/plate/experimental effects [15]. This is achieved by three main steps on a per-gene basis. In a first step, the gene signal is corrected for possible technical noise. The mean background signal, obtained from RNA sample free wells, is subtracted from the raw median fluorescence intensity values. In a second step, the relative gene expression is calculated by dividing the background corrected gene signals with the geometric mean of the housekeeping genes (HKG), which are taken along in the experiment. Finally, in a third step, fold changes are computed by dividing the relative expression value by the median relative expression value from the untreated samples (i.e. negative control). The proposed three step signal transformation approach will, however, only be effective in reducing technical/experimental related variability, when applied to high-quality and stable signals. To the best of our knowledge, clear instructions on how to assess the data quality are currently lacking, while most researchers perform the transformations in a local spreadsheet environment or by means of manual calculation.

Against this background we present experimental data tested in dose response and introduce a newly developed open source available R based shiny application: QGprofiler, that allows for proper QuantiGene® Plex 2.0 assay optimization, choice of HKG and data pre-processing from raw gene expression to normalized FC values including appropriate QC metrics. In addition, we propose a way to assess cytotoxicity and introduce a dose response FC. QGprofiler is available at https://qgprofiler.openanalytics.eu/app/QGprofiler and will accept a 96 multi-well plate format in dose response.

## Methods

### Experimental conditions

In order to assess the minimal number of beads, the stability of the HKG and optimize the data analysis flow, data from 304 samples (i.e. samples treated with different compounds in different dose ranges) were generated from five independent QuantiGene® runs (Table 1). Cell lysates were prepared according to manufacturer’s instructions, using the QuantiGene® Sample Processing Kit (Cultured Cells), while the QuantiGene® assay was run on a Luminex FlexMAP® 3D platform, following the QuantiGene® 2.0 Plex user manual, distributed by ThermoFisher Scientific [15].

**Table 1:** Overview of the different experimental settings with number of samples, compounds, genes, HKG and background used to assess the ideal number of beads, linearity of HKG signal and overall QGprofiler data flow. Where samples are either vehicle or compounds tested in multiple dose in a particular background

Experiment		# Samples	Total # Genes	# HKG	# Compounds	Background
Bead Number	Dataset 1	46	14	3	2	Xenograft A
	Dataset 2	6	14	3	1	Xenograft B
	Dataset 3	132	18	3	19	Cell line C
Linearity of HKG	Dataset 4	60	26	26	0	Cell line C
Data Analysis Flow	Dataset 5^a^	60	20	2	10	Cell line C

^a^data set is provided to explore the QGprofiler application

### Bead number

Three independent datasets, together measuring 29 unique genes with an average > 50 beads per gene/well were used to define the minimal number of beads, needed to produce stable median fluorescence intensity values (Table 1). A total of 10, 20, 30, 40 and 50 fluorescent signals (i.e. beads) were subsampled per gene and per well and their median fluorescence intensity value was calculated. These median values were compared to the median fluorescence intensity, produced by the entire bead set, assumed to be the golden standard (eq.1). The subsampling procedure was repeated 100 times for each gene/well combination.

1 $$ \%{dev}_{ij}=\frac{\overset{\sim }{X_{ij}^{\ast }}-{X}_{ij}}{X_{ij}}\ast 100 $$

$$ with\kern0.5em \overset{\sim }{x_{ij}^{\ast }}\  the\ median\ of\ the\ resampled\ fluorescent\ signal\ and $$

$$ {x}_{ij}\  the\ reported\ median\ fluorescent\ signal\ using\  all\  available\ beads $$

$$ for\ each\ well\ j\  and\ gene\ i $$

The obtained deviation percentages were subsequently combined across the different wells per gene, by taking the overall mean. These values were plotted in function of the number of beads (i.e. 10 to 50) and piece-wise linear regression was applied. This regression model enabled the determination of a gene specific bead number threshold above which adding beads does no longer significantly affect the stability of the deviation percentages.

### Linearity of HKG signal

One data set, measuring 26 HKG across 60 samples, with an average > 50 beads per gene/well was used to assess the limit of quantification (LOQ) (Table 1). The linearity of the background corrected signals as a function of the cell counts was investigated to define and set the LOQ. Both 5 and 10 SD above the mean background median fluorescence intensity values were considered as potential LOQ thresholds, as suggested by ThermoFisher Scientific [15].

### Data analysis flow

One dataset, measuring 20 unique genes and 2 HKG across 60 samples (10 compounds which 6 doses each) in one cellular background with an average > 50 beads per gene/well was used to demonstrate the data analysis flow, ranging from the raw gene expression value to the FC computation, including assessment of HKG stability (Table 1). To correct for experimental differences, the relative gene expression was calculated, as the ratio of the background corrected values versus the geometric mean of the HKG. Finally, FC values were computed by dividing the relative expression value of each sample by the median relative expression value from the untreated samples (i.e. negative control). Stability of the HKG was assessed at the FC level. HKG that fell outside the [0.8; 1.2] FC range were removed from the analysis, until the FC of the remaining HKG met this FC range criterion.

### Dose response modeling

Dose response curves were fitted to the FC values by means of the drm() function, available in the R package drc [16]. Let *y* denote the observed FC value corresponding to dose value *x* (*x* ≥ 0). The mean of *y*, *E(y)*, is characterized using the model function *f* depending on dose *x*:

2 $$ E(y)=f\left(x,\left(b,c,d,\overset{\sim }{e}\right)\right)=c+\frac{d-c}{1+\mathit{\exp}\left(b\left(\mathit{\log}(x)-\overset{\sim }{e}\right)\right)} $$

This model is a log-logistic model, sometimes referred to as a Hill model, which describes a typical s-shape for the mean response. The slope parameter *b* denotes the steepness of the curve, (i.e. Hill slope), while *c* and *d* are the asymptotes of the response and ẽ refers to the logarithm of the effective dose (ẽ = log(AC50)) [16]. A parameter constraint (i.e. *b* > 0) was imposed to ensure that parameters *d* and *c* refer to the plateaus of the dose-response curve at dose zero and maximal dose, respectively, independent of the increasing/decreasing nature of the dose-response curve. As such, setting *d* = 1 fixed the plateau at dose zero for all dose-response curves at FC = 1 (i.e. mean FC value of negative controls). In addition, a second parameter constraint (i.e. c > 0) was imposed to ensure that FC values could not be modelled in the negative region of the response.

The resulting model is referred to as a three-parameter log-logistic (3PL) model with model parameters *b* > 0, *c* > 0 and *ẽ* estimated from the data. A model fitting scheme was applied to obtain a final model fit for each compound/gene combination. If all response FC values were observed within the [0.8; 1.2] FC range, the response was regarded as uninformative [15] and a constant fit was applied. If at least 1 FC value fell outside the [0.8; 1.2] FC range, three different models were fitted, including (1) a constant fit, (2) a 3PL dose response model as described above and (3) a weighted version of the 3PL dose response model as described above, down weighting high FC values to address variance heterogeneity in the response (i.e. ${w}_i=\frac{1}{FC_i^2}$). The final model was chosen using the Akaike information criterion (AIC).

Finally, the absolute AC50 values were extracted as potency estimates, using the model parameters of the most optimal fit by the ED() function of the drc package [16]. These absolute AC50 values correspond with a FC of 0.5 or 1.5 for a decreasing or increasing dose response curve respectively. The absolute AC50 values were chosen over the relative AC50 values, since the former are more robust against bias in the estimation of the second asymptote which is not always reached in the tested dose range.

### Input files QGprofiler

QGprofiler requires the input of two files. In a first .xls(x) file, a template should be provided which maps each well to its specific experimental condition (an example template file can be found in Additional file 4). This file should contain two sheets named: *Plate* and *HKG.* If multiple plates need to be processed at the same time, the file needs to contain an additional third sheet: *Rawdat*a. The *Plate* sheet specifies for each well the following variables: compound, concentration and cell count, timepoint and cell line. A short description for each of these variables is provided in Additional file 3: Table S1. Wells for which none of the variables are annotated, will be discarded when loading the raw data. Variables that are never annotated are assumed to be constant across the plate and will not be considered during the analysis.

The text provided in cell A1 generates the title of the data set and is used throughout the analysis (e.g. graph titles, file export names). The *HKG* sheet tabulates the housekeeping genes, which should be listed in column A. The gene names must correspond to the gene names used in the raw QuantiGene® data file. The *Rawdata* sheet contains the name of the raw data file that needs to be merged with the template in cell A1. A second .txt or .csv file contains the name of the raw QuantiGene® data file, generated by the Luminex FlexMAP® 3D platform (Additional file 5).

## Results

**Fig. 1:**
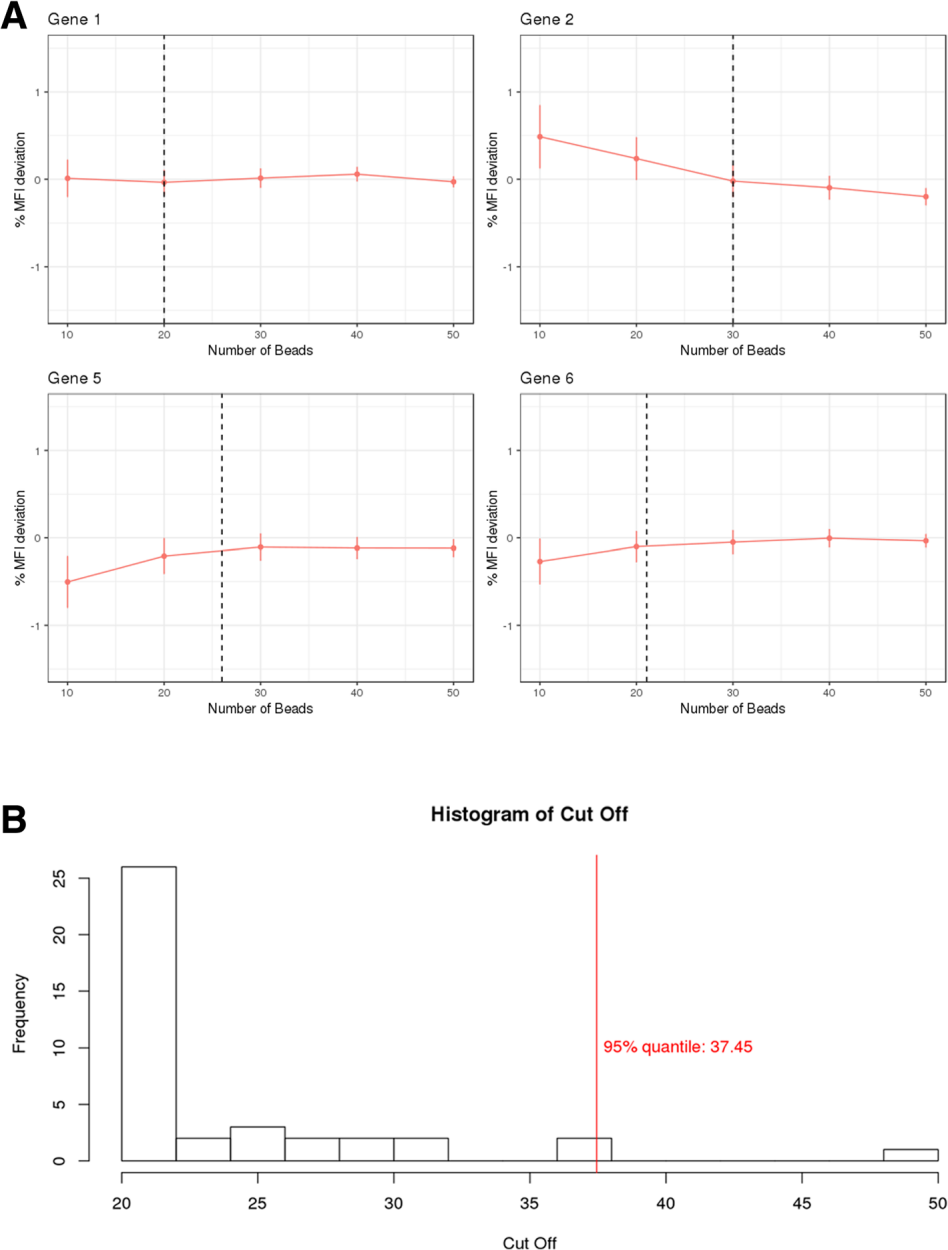
**a** Percentage MFI deviation from the observed full bead dataset (≈ 60) as a function of bead number subsamples (10, 20, 30, 40 and 50) for gene 1, 2, 5 and 6. The vertical dotted line indicates the piece-wise regression cutoff point. **b** Distribution of the bead number thresholds, above which the percentage MFI, relative to the entire bead population MFI is no longer affected, defined by piece-wise linear regression. The 95th % bead number quantile is indicated with a vertical dashed line

**Fig. 2:**
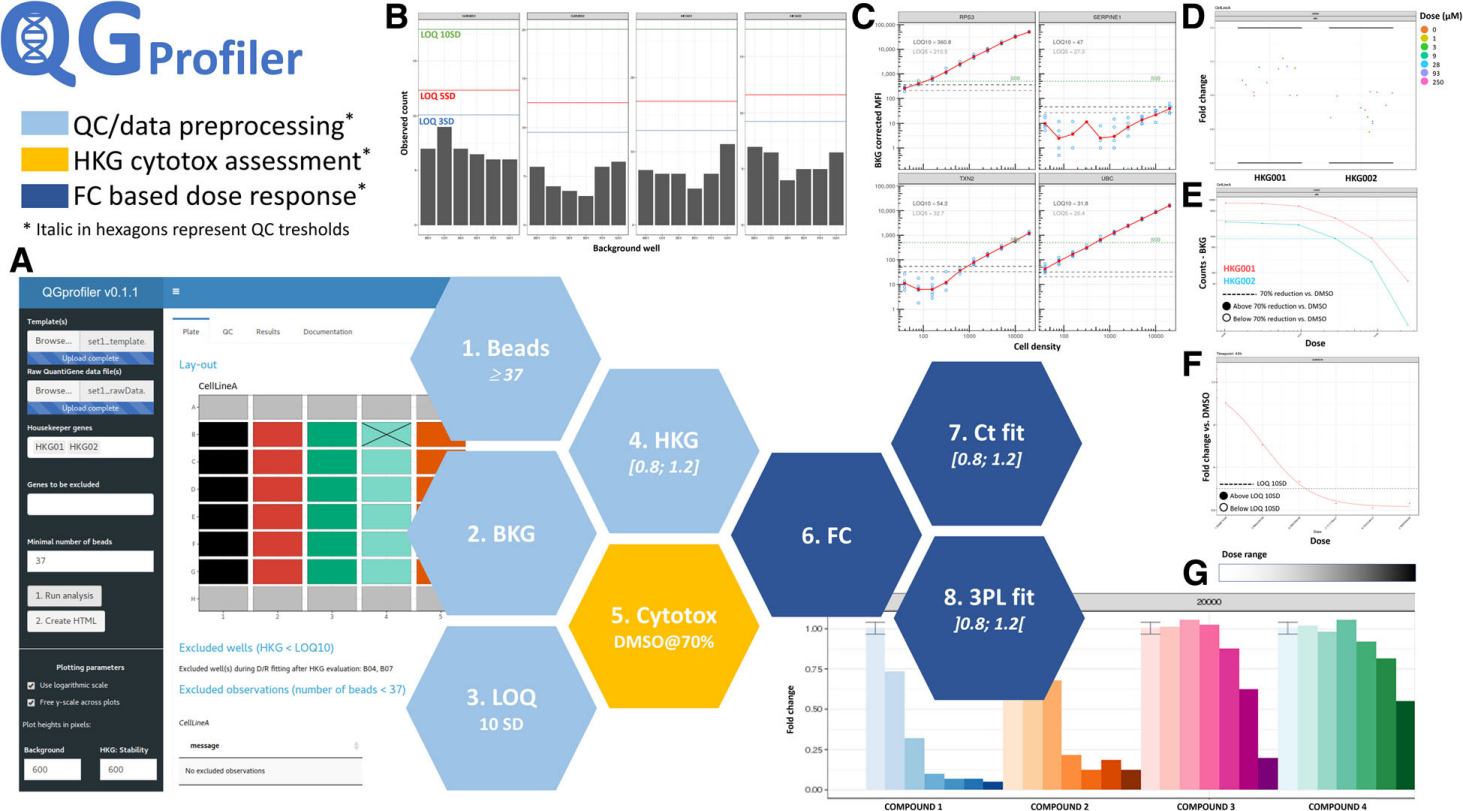
Overall QGprofiler quality assessment and data (pre-)processing analysis flow represented by the different hexagons and illustrated by different QGprofiler’s screenshots. **a** QGprofiler interface, with required user-supplied inputs on the left panel and output on the right panel. Output is organized in three: general plate information (Plate tab, shown); various quality control steps (QC tab); and dose response modelling results and export options (Results tab). The Documentation tab provides extensive information on QGprofiler usage. The Plate tab provides a visualization of the experimental setup of the plate as well as information on which well/genes are disregarded based on minimal number of beads or LOQ_10_. **b** Quality control of background signals. MFI counts for each background well are depicted across all measured genes. Blue, red and green lines indicate the limits of quantification, set at 3, 5 and 10SD (LOQ_3_, LOQ_5_ and LOQ_10_). These are calculated as the mean MFI value across all background wells plus three, five or 10 standard deviations of the background, respectively. **c** Quality control of background corrected signals in function of cell count for a series of HKG (more HKG were tested, data not shown). As a rule of thumb, LOQ_10_ can be regarded as the lower limit of HKG signal linearity. **d** Quality control on HKG stability on a 20% FC range, expressed per HKG, cell density, exposure time and/or cell line. **e** Dose response relationship of background corrected HKG MFI values, indicating cell cytotoxicity. The horizontal dashed lines indicate the background corrected MFI threshold based on the negative controls for the corresponding HKG. **f** FC barplot on a per-gene basis, across the different compound concentrations in which each compound dilution series is plotted with a separate gradient color. Across this specific experiment some compounds show almost no effect, while others reveal a clear dose-response relationship. **g** Example of the final dose response 3PL model for a gene of interest in cells exposed to a compound of interest. Dotted horizontal line represents the absolute AC50 level

Typically, QuantiGene® raw data are formatted as median fluorescence intensity values across individual beads. The individual bead numbers have been reported to range from 50 to 100 beads per gene per well, while it is generally recommended to have an average of 50 bead counts per gene. Factors such as sample viscosity, washing steps throughout the assay and possible bead carryover across wells, can cause a drop in the actual number of beads per gene/well which might destabilize the reported median fluorescence intensity values. The percentage by which the median fluorescence intensity values for different bead numbers deviate from the entire bead set generated median fluorescence intensity values, is represented per gene across the different wells in Fig. 1a. The bead number threshold distribution, above which the median fluorescence intensity value, relative to the median fluorescence intensity values for the entire bead population is no longer affected, defined by piece-wise linear regression, is plotted in Fig. 1b. The 95th percentile of this distribution corresponds to 37.45 beads per gene and well, indicating that 5% of the genes will return a less stable median fluorescence intensity value when bead numbers drop below 37 (Fig. 1b). Based on this assessment it was decided to set the bead number threshold in QGprofiler by default at 37 beads per gene and well. As a result, QGprofiler will automatically discard raw median fluorescence intensity values that were obtained from a total number of beads < 37 and will subsequently list all discarded gene/well combinations in its QC tab (Fig. 2a). Nevertheless, this parameter is made flexible and can be adjusted depending on the risks one is willing to take dependent on the stage of the project, as the majority of genes are already stable from 30 beads onwards. Once these wells with insufficient bead numbers are removed, a background correction is performed. The latter subtracts the mean background value from the raw median fluorescence intensity values in a gene-specific manner. QGprofiler provides the possibility to visually inspect all background signals, enabling the identification of deviating background wells on a per gene basis (Fig. 2b). At this stage, deviating wells can be removed from the analysis by removing them from the template file. Negative signals after background correction are not accepted and will be set to zero.

Figure 2c represents the mean background corrected median fluorescence intensity value across negative control wells for a selection of HKG in function of cell count. The mean background corrected median fluorescence intensity values across all background wells plus 5 and 10 SD of the background signal were plotted as potential LOQ thresholds. It is clear from Fig. 2c, that the mean of the median fluorescence intensity values across the background wells plus 10 SD of the background signal can be used as LOQ. Indeed, above 10 SD, the background corrected median fluorescence intensity signal of most HKG falls within the linear range and can thus be used for further analysis. All wells for which at least one HKG mean background corrected median fluorescence intensity drops below LOQ_10_, will automatically be disregarded by QGprofiler and will be listed in its QC tab. The LOQ_10_ based data removal step is only performed for the HKG, since the disease relevant genes that fall below LOQ_10_, might still contribute to the dose response fitting. To properly describe the background well median fluorescence intensity distributions and thus the gene specific LOQ_10_ thresholds, it is advised to increase the number of background wells from 3 to 6, contrary to what is suggested by ThermoFisher Scientific [15].

**Fig. 3:**
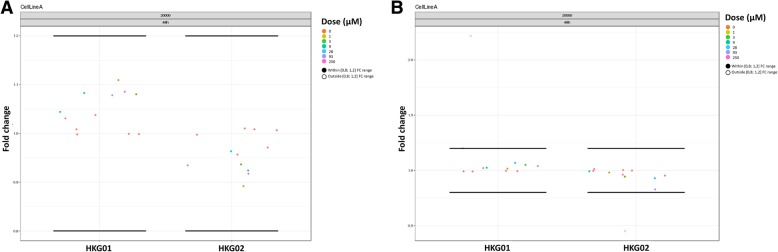
QGprofiler’s HKG quality control chart as a function of the experimental conditions. In the current experiment, the FC data is plotted as a function of the cell density, compound dose and exposure time. Horizontal lines represent the HKG FC stability acceptance threshold of 20%. **a** Example of a HKG that falls within the stability threshold and which can be used for downstream analysis. **b** Example of a HKG that falls outside the stability threshold

**Fig. 4:**
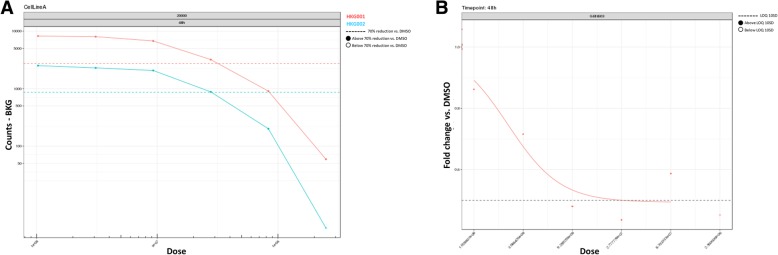
**a** Dose response relationship of background corrected HKG MFI values. The horizontal dashed lines indicate the background corrected MFI threshold based on the negative controls. Compound treatments crossing the threshold in a dose dependent manner is indicative for cytotoxicity. **b** Example of a dose response relationship on FC for a gene of interest after treatment with a specific compound, where normalization failed due to instable HKG. Over correction occurred at the second highest concentration, though not immediately visible from the FC plot (see Fig. 3b) but signals drop far below the negative control levels (**a**). This compound will be indicated as potential cytotoxic and results should be interpreted with care

Once the technical noise is removed, the gene signal is normalized against the HKG, which are chosen because of their stability in the required experimental conditions during assay optimization. This normalization allows for correction of experimental differences and is performed by calculating the relative expression, which is the ratio of the background corrected median fluorescence intensity values for a certain gene versus the geometric mean of the background corrected median fluorescence intensity values for all HKG. If the HKG are not stable across different experimental conditions, they may over or under correct the gene signal. Hence, it is key to define a proper set of stable HKG using the appropriate assay conditions, prior to the drug discovery campaign and to keep this set constant during the campaign itself. A HKG is said to be stable if its corresponding FC values lie within the [0.8, 1.2] FC interval, (i.e. accepting a 20% variability [15]). QGprofiler provides metrics and visuals to inspect the stability of the HKG across the experimental conditions (Figs. 2d, 3). When correctly chosen during assay optimization, HKG are not expected to fall outside the [0.8, 1.2] interval. However, it is regularly observed during drug discovery campaigns that HKG deviate from the 20% FC range for some compounds, which may be indicative for compound induced cytotoxic effects. It has been shown (data not provided), that the 20% FC interval is even not sensitive enough to assess compound induced cytoxicity. Hence, in addition to the FC assessment, QGprofiler investigates the background corrected HKG dose response plots to flag the cytotox potential of a compound (Figs. 2e, 4). If the background corrected median fluorescence intensity value for one or more HKG drop below a 70% reduction of the mean background corrected median fluorescence intensity value across all negavtive control wells, in a dose dependent manner, the compound*dose condition is marked as cytotoxic in QGprofiler. These conditions (dose and compound) will be listed in the QC tab and the final efficacy table provided by QGprofiler (Figs. 2a, 3b) and these compounds are recommended to be followed-up in more specific assays. While the normalization procedure using HKG has some pitfalls, it also has some additional strengths of flagging potential cytotoxic compounds (Fig. 4). This normalization step is most crucial for a proper data transformation and requires careful investigation of all HKG quality metrics that are provided in QGprofiler both during assay optimization as well as during the screen itself (Fig. 2).

FC values are subsequently computed by taking the ratio of the relative expression of each gene versus the median relative expression for the same gene across the negative control wells. Since the negative controls are key to define both FC and cytotoxicity (i.e. 70% drop versus median negative control) it is imperative to include at least six negative control wells, similarly as with the background wells, for a proper distribution estimate. QGprofiler provides the possibility to inspect the negative control values across the different genes as well (plots not shown).

Once the quality of the different metrics computed during data transformation from raw median fluorescence intensity to FC, has been evaluated, the gene specific dose response (i.e. FC) relationship can be modelled in QGprofiler (see Material and Methods) (Fig. 2g). Although all curves will start at a fixed FC value of 1, it is often challenging to estimate how the curves might end, since the maximal effect is not always reached in the investigated dose range. Hence, QGprofiler will tabulate the absolute AC50 value instead of the relative AC50 value. Absolute AC50 values correspond to the concentration where a FC of 0.5 or 1.5 is reached, depending on the sign of the Hill slope. This metric is not, in contrast to the relative AC50, dependent on the estimation of the maximum effect and will therefore be more robust against incomplete curves. Next to the absolute AC50 (log and original scale), QGprofiler will also tabulate the Hill slope, maximal effect, associated standard errors and FC at maximal concentration, as extracted from the final model, selected based on the AIC criterion. In addition, a cytotoxicity flag will be shown when the background corrected median fluorescence intensity for a HKG drops below the cytotoxic threshold as explained above.

## Discussion

The user friendly, open source available R based shiny application, QGprofiler, is developed to support drug discovery campaigns. As such, it is currently used as a primary screening tool in multiple lead optimization projects within J&J, both within the field of infectious diseases and oncology. In this context, entire compound libraries/series are tested against a gene signature of choice in search for new disease relevant chemical entities both in in-vitro as well as in-vivo backgrounds representative for the disease. As proposed by ThermoFisher Scientific [15], raw gene values are transformed to FC values which are subsequently modelled in dose response to extract gene specific AC50 values when compounds are profiled in dose response otherwise FC are compared across conditions. To be successful and select the most promising chemical candidates, it is, however, crucial that FC and downstream efficacy estimates remain stable and comparable across different experimental runs. The latter is achieved in QGprofiler, by introducing quality control steps along the entire data transformation and analysis process. These steps include (1) minimal bead number assessment that is needed to generate stable raw fluorescent signals, (2) stability control of the background signal, (3) selection and stability control of the HKG and (4) quality assessment of the negative controls. In addition, QGprofiler offers the possibility to perform dose response modelling and makes use of the HKG background corrected signals to infer and flag cytotoxicity of compounds. The latter is only possible when HKG are properly chosen during the assay optimization, prior to the drug discovery campaign. A series of stable HKG have been suggested by Thermofisher, including ACTB, ATP6V1A, B2M, GADPH, GUSB, HMBS, HPRT1, LDHA, PGK1, POLR2A, PPIA, PPIB, RPL13A, RPL19, RPL32, RPLP0/Arbp, RPS3, RPS18, RPS20, RPS23, RPS29, TBP, TFRC and TXN2. However, depending on the experimental conditions, at least some of these HKG (i.e. PPIB, RPLP0, LDHA, RPS20, B2M, GADPH and RPL13A) can go outside of the 20% FC variability range (Additional file 1: Figure S1). Hence, it should be stressed that assay optimization across a wide range of experimental conditions (e.g. cell density, exposure time, dose range, compound class) is a crucial pre-requisite for success.

The latter falls outside the scope of this publication. However, these assay optimization assessments are nonetheless available in QGprofiler. For instance, QGprofiler allows the user to define the most optimal cell density for a series of compound doses and/or times of exposure as a function of the linearity of the QuantiGene® signal (Additional file 2: Figure S2).

After appropriate pre-processing of the raw gene signals and FC modelling, QGprofiler will generate AC50 values. These gene specific AC50 values are the primary parameters of interest during large scale drug discovery campaigns, as they allow to pin point and rank the most promising drug candidates within the extended screened chemical space. However, it is important to assess the standard errors that are associated with the AC50 values to allow for a proper AC50 assessment and thus determination of its (un) certainty. When the standard errors on log10 AC50 are larger than 0.3 (i.e log10(2)), it is advised to repeat the experiment, increasing the number of replicates and/or revising the concentration range. These estimates are indicated with a ~in the final results table. Additionally, it must be mentioned that QGprofiler will only generate absolute AC50 values, as they are more robust against incomplete curves compared to their relative AC50 counterparts. Nonetheless, the absolute AC50 estimate is based on a 3PL model fit, and one would thus ideally need at least two concentrations beyond the absolute AC50 value to retrieve unbiased estimates.

**Fig. 5:**
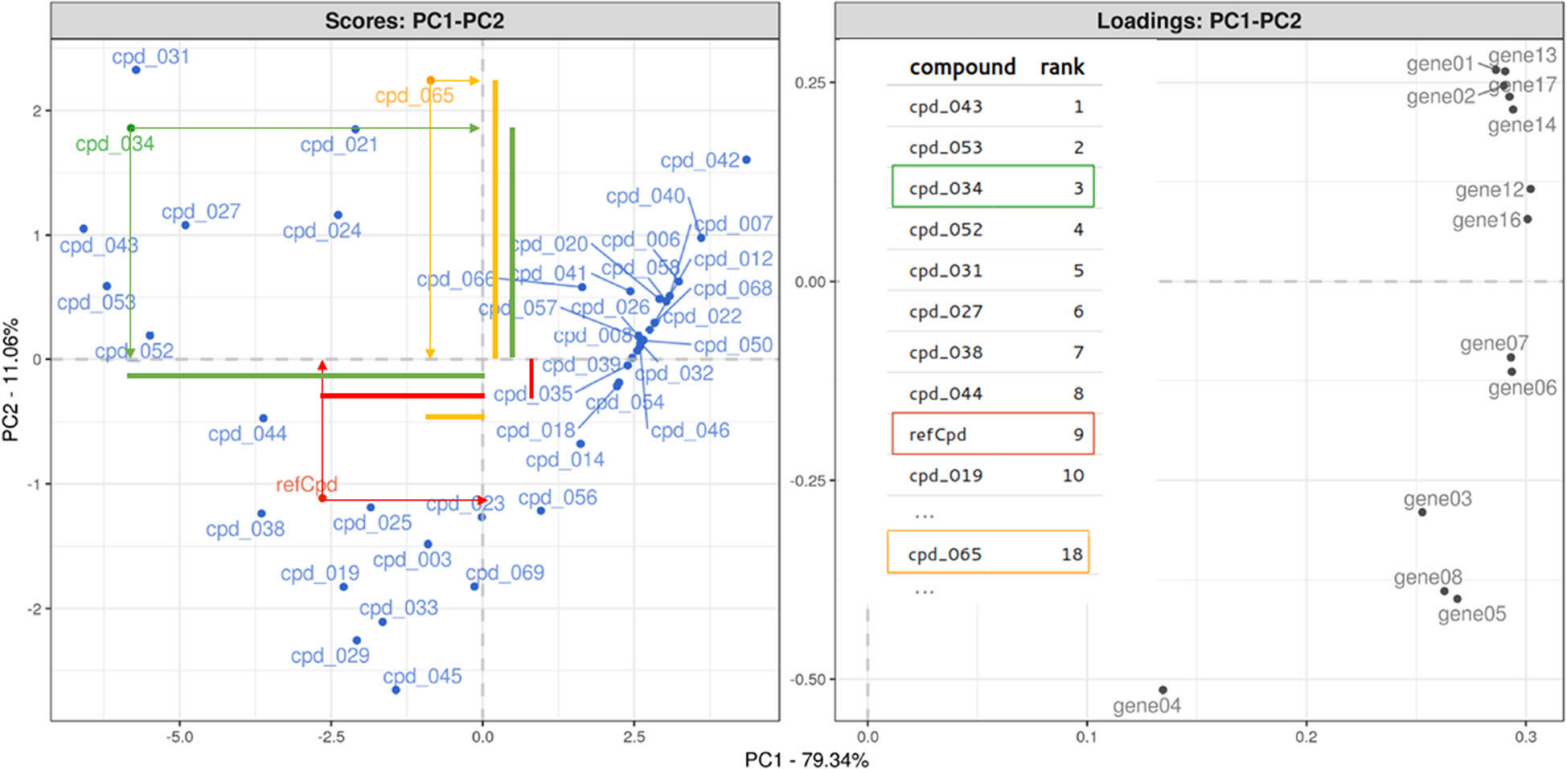
PCA based compound ranking with superimposition of the gene signature. The first principal component is mainly driven by the disease genes. Compounds can be prioritized based on PCA weighed distances as illustrated for cpd_034 (rank 3) and cpd_065 (rank 18), with compounds on the left of the refCpd having some promising transcriptional signal for the target related gene signature

While the QuantiGene® Plex 2.0 assay allows measuring up to 80 genes simultaneously, ranking compounds on all these potency estimates becomes challenging. Instead of looking at gene-specific ranks, one could move to consensus ranking techniques (R-package ConsRank [17]) which is based on median ranks across the genes according to the Kemeny’s axiomatic approach [18]. Alternatively, supervised and/or unsupervised multivariate projection/ordination techniques (e.g. PCA, CDA, TSNE) can reduce the dimensionality and allow for compound ranking in a reduced space while retaining most of the transcriptional information. Such techniques extend the strength of the QuantiGene Plex® 2.0 assay, in a multidimensional transcriptional decision making/ranking compound space. Figure 5 represents such an analytical approach in lead optimization stage for an oncology related target where compounds are ranked on transcriptional signatures. Starting from the FC dose response model (Fig. 2f), the absolute AC50 value is extracted for each gene/compound combination and used to ordinate all compounds along the first principal component axes, which captures 90% of the transcriptional signal. The genes that drive the observed compound ordination are superimposed. Based on the weighed PCA score distances between the compound(s) and a reference compound, all screened compounds can be sorted, ranked and prioritized, as their distance score is a measure for their desired transcriptional effect.

Currently we are expanding QGprofiler to accommodate both 96 and 384 multi-well plate formats. In addition, QQprofiler will be able to process data from (a) QuantiGene® singleplex, (b) QuantiGene® multiplex and (c) QuantiGene® to accommodate the throughput increment in our drug discovery campaigns. As such a single gene is exposed to a single compound, a series of genes are exposed to a single compound and a series of genes are exposed to a series of compounds in a single well respectively. All these applications can be performed in both 96 and 384 well format and rely on the same bDNA technology. As such, the same normalization and transformation processes as described above, all available in QGprofiler, can be used to assure accurate data quality in single, multi and plex on plex QuantiGene® experimental settings, to be used in further downstream analysis.

## Conclusions

The transcriptional profiling QuantiGene® Plex 2.0 platform (ThermoFisher Scientific) can be used in a drug discovery setting, since it allows multiplexing in a single well of a 96 or 384 multi-well plate in which cells are exposed to entire chemical libraries. Data interpretation follows a three-step normalization/transformation flow in which raw median fluorescent gene signals are transformed to fold change values with the use of proper housekeeping genes and negative controls. Clear instructions on how to assess the data quality and tools to perform this analysis in high throughput mode are, however, currently lacking. We have developed a user-friendly open source R based shiny application, QGprofiler to address these needs in a series of control and analysis steps. These steps include the (1) minimal bead number assessment to obtain stable raw fluorescent signals, which has been set to 37 beads per well and gene, (2) stability control of background signal and determination of the gene specific lower limit of quantification, (3) selection and stability assessment of HKG based on FC distributions within the FC range of 0.8 and 1.2, (4) geometric mean normalization with the remaining HKG and computation of relative gene expression values, (4) quality assessment of the negative controls, (5) dose response modelling at the FC level and (6) possibility to infer cytotoxicity on the HKG background corrected signals versus the negative control distribution. QGprofiler is available at: https://qgprofiler.openanalytics.eu/app/QGprofiler

## Additional files

Additional file 1: **Figure S1.** FC based stability of a series of commonly used HKG in function of cell density, (i.e. 500 and 2500 cells/well) keeping all other parameters constant. (PNG 187 kb)

Additional file 2 **Figure S2.** Selection of optimal cell density based on the linearity of the background corrected signal for each of the genes. Optimal condition is in the mid cell densities. At the high-end saturation of the signal is observed, while at the other end the signal drops below the LOQ10 for some genes. (PNG 64 kb)

Additional file 3: **Table S1.** Experimental variables to be annotated in QGprofiler template input file. (DOCX 16 kb)

Additional file 4: QGprofiler template plate layout file. (XLSX 9 kb)

Additional file 5: QGprofiler raw data input file. (CSV 28 kb)

## Data Availability

https://qgprofiler.openanalytics.eu/app/QGprofiler

## Abbreviations

AIC: Akaike Information Criterion
bDNA: Branched Chain DNA
CDA: Canonical Discriminant Analysis
FC: Fold Change
HKG: Housekeeping Genes
LOQ: Limit of Quantification
LOQ_10_: Limit of Quantification at 10SD
MFI: Median Fluorescent Intensity
PCA: Principal Component Analysis
SAPE: Streptavidin Conjugated R-Phycoerythrin
TSNE: T-Distributed Stochastic Neighbor Embedding
