# Supplementary figures and images for: Analyzing magnetic bead QuantiGene® Plex 2.0 gene expression data in high throughput mode using QGprofiler
Source: BMC Bioinformatics. 2019 Jul 8;20:378. doi: 10.1186/s12859-019-2975-2 (PMC6615108; doi:10.1186/s12859-019-2975-2)

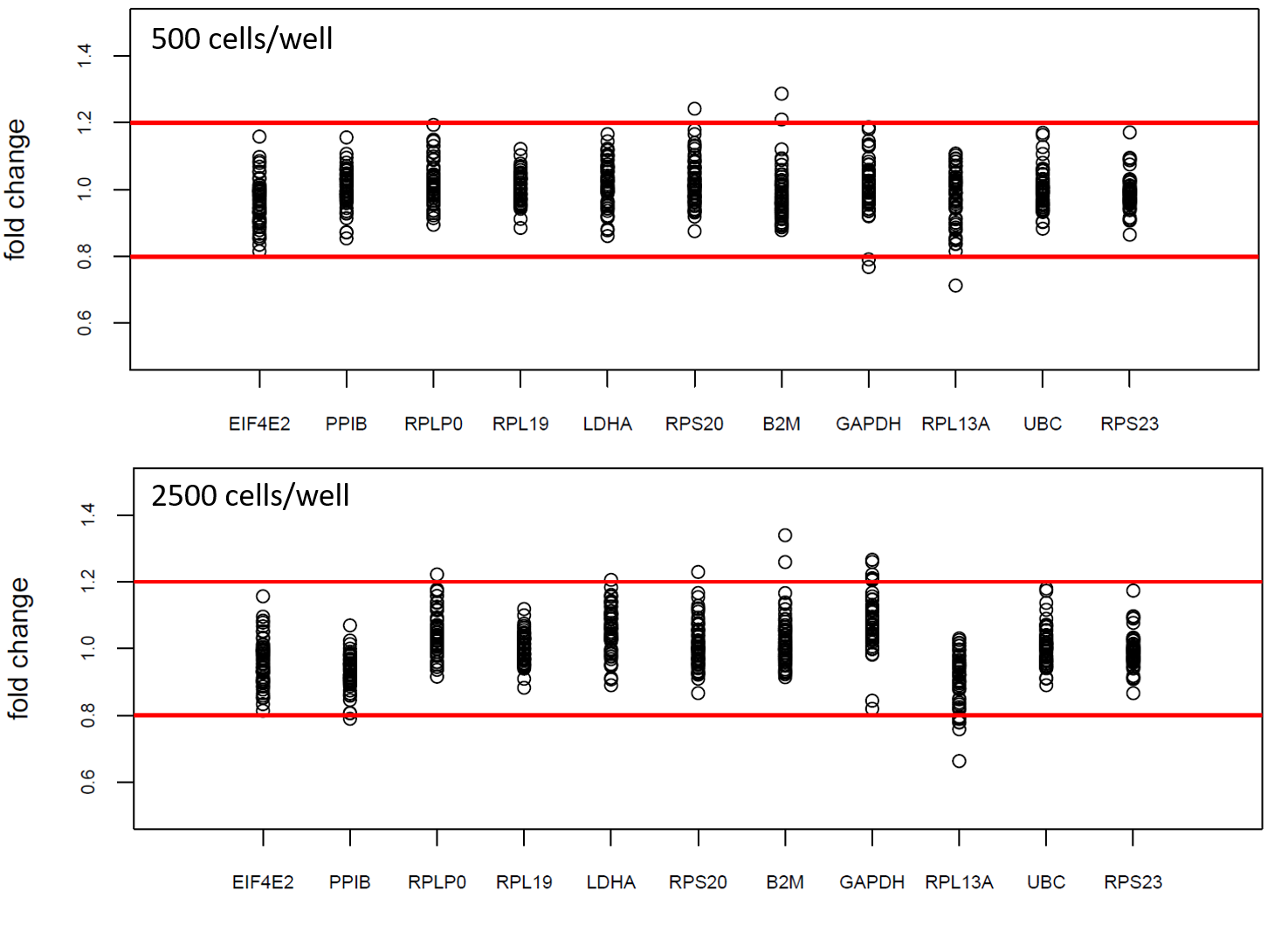

Supplement: Supplementary file 1 — Figure S1. FC based stability of a series of commonly used HKG in function of cell density, (i.e. 500 and 2500 cells/well) keeping all other parameters constant. (PNG 187 kb) [file 12859_2019_2975_MOESM1_ESM.png]

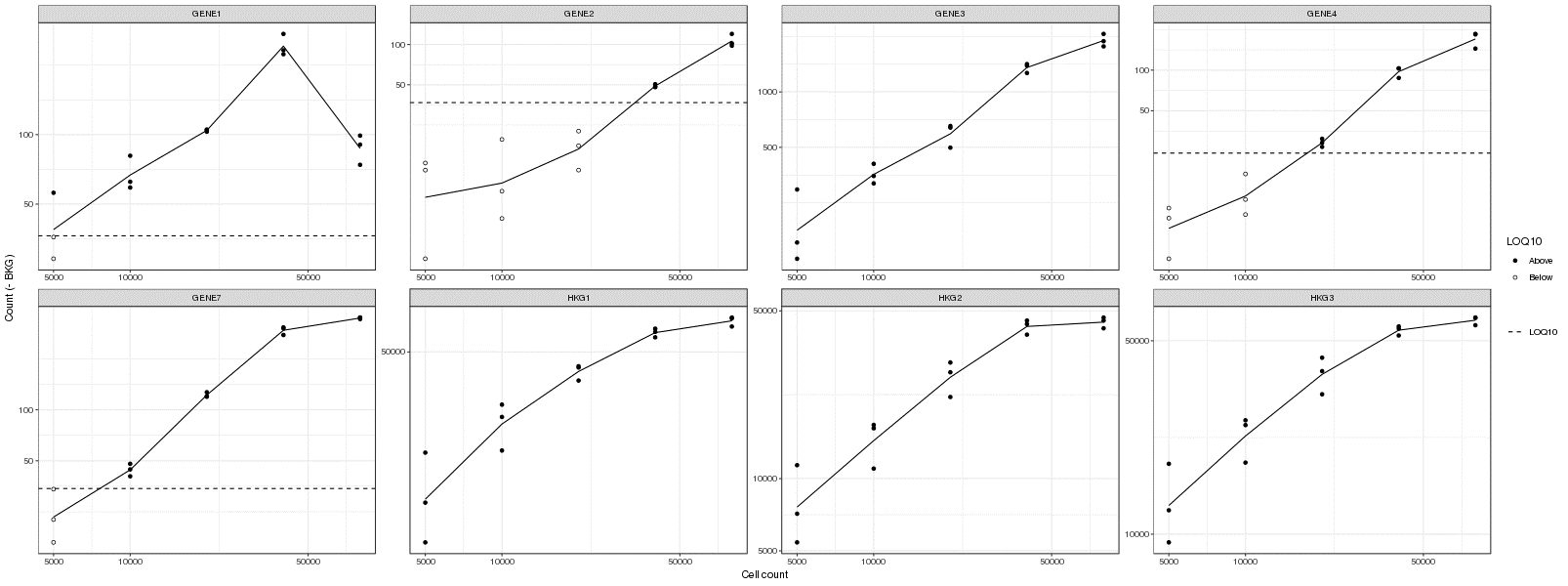

Supplement: Supplementary file 2 — Figure S2. Selection of optimal cell density based on the linearity of the background corrected signal for each of the genes. Optimal condition is in the mid cell densities. At the high-end saturation of the signal is observed, while at the other end the signal drops below the LOQ10 for some genes. (PNG 64 kb) [file 12859_2019_2975_MOESM2_ESM.png]
