# Supplementary material for: Analyzing magnetic bead QuantiGene® Plex 2.0 gene expression data in high throughput mode using QGprofiler
Source: BMC Bioinformatics. 2019 Jul 8;20:378. doi: 10.1186/s12859-019-2975-2 (PMC6615108; doi:10.1186/s12859-019-2975-2)
Supplement: Supplementary file 3 — Table S1. Experimental variables to be annotated in QGprofiler template input file. (DOCX 16 kb) [file 12859_2019_2975_MOESM3_ESM.docx]

**Table S1** Experimental variables to be annotated in QGprofiler template input file

|  | Description |
| --- | --- |
| **Concentration** | Sample concentration in µM  Tick ‘Dose values on mg/kg scale’ box in case concentrations are in mg/kg  Must be annotated for wells that are used; should not be filled in for background wells |
| **Cells** | The number of cells |
| **Time point** | Time point indicator |
| **Cell line** | The cell line used |
| **Compound** | The compound name  Do not use spaces; use ‘Background’ for background wells and ‘DMSO’ or ‘Vehicle’ for negative control wells |
